# Prevalence and Radiological Evaluation of Primary Molar Infraocclusion in a Group of Turkish Children: A Retrospective Study

**DOI:** 10.3390/children13020229

**Published:** 2026-02-06

**Authors:** Ecem Elif Çege, Neşe Akal

**Affiliations:** 1 Department of Pediatric Dentistry, Faculty of Dentistry, Karabük University, 78050 Karabük, Turkey; 2 Department of Pediatric Dentistry, Faculty of Dentistry, Gazi University, 06490 Ankara, Turkey

**Keywords:** dental anomalies, infraocclusion, prevalence primary molars

## Abstract

**Background**: This study aimed to determine the prevalence of infraocclusion in primary molars, its distribution according to age, gender, and location, dental anomalies associated with infraocclusion, radiographic findings related to infraocclusion, and treatments applied to affected teeth. **Methods**: A total of 8452 digital panoramic radiographs of children aged 7–11 years (2019–2022) were retrospectively evaluated for infraocclusion of primary molars. Radiographs were assessed by calibrated examiners, and infraocclusion severity was classified according to the Brearley and McKibben system. The presence of permanent successors, applied treatments, and associated dental and radiographic findings were recorded. Data were analyzed using descriptive statistics and chi-square tests (SPSS 28.0). **Results**: Infraocclusion was most frequently observed in mandibular second primary molars and was most prevalent at the age of 9. The majority of cases were mild (49.5%), followed by moderate (29.8%) and severe (20.7%). A significant correlation was found between infraocclusion severity and radiographic findings such as adjacent tooth tipping, supraeruption of opposing teeth, and eruption disturbances of permanent successors (*p* < 0.05). A statistically significant relationship was also observed between infraocclusion severity and treatment modality (*p* < 0.05). As infraocclusion severity increased, restorative treatment decreased and extraction rates increased. In 10.3% of cases, no permanent successor was present beneath the affected tooth. Dens invaginatus and hypodontia were the most commonly associated dental anomalies. The overall prevalence of infraocclusion was 3.6%, with no significant gender difference (*p* > 0.05). **Conclusions**: Although infraocclusion is relatively uncommon in primary molars, the present findings highlight its frequent associations with dental anomalies and variations in permanent successor presence. These results emphasise the importance of careful radiographic evaluation and follow-up in children with infraoccluded primary molars. Future longitudinal studies are needed to better understand the aetiology and progression of infraocclusion.

## 1. Introduction

Infraocclusion is defined as a condition in which the occlusal surfaces of affected teeth lie below the occlusal plane or the occlusal surfaces of adjacent teeth. While the affected tooth remains retained, eruption and alveolar growth continue in adjacent areas. Infraocclusion severity ranges from mild infraocclusion to severe cases where the tooth is completely submerged below the alveolar bone [1]. Although terms such as ankylosis, submerged teeth, incomplete eruption, and secondary retention are frequently used interchangeably with infraocclusion in the dental literature, they represent different conditions with distinct etiologies, pathophysiological mechanisms, and effects on alveolar bone development [2,3].

Primary teeth, also known as deciduous teeth, play a critical role in guiding permanent tooth eruption and maintaining space within the dental arch. Infraocclusion prevalence in primary molars varies among different populations, ranging from 2.8% to 41.8% (Table A1, Appendix A) and appears equally distributed across genders, reaching peak prevalence around 8–9 years of age [4,5]. Infraocclusion typically affects mandibular teeth bilaterally, often involving multiple primary molars simultaneously. However, uncertainty remains regarding whether the first or second molars are most commonly affected (Table A1, Appendix A) [6].

The aetiology of infraocclusion is multifactorial and remains incompletely understood. Ankylosis, defined as fusion between the tooth root and alveolar bone following periodontal ligament trauma or inflammation, is the most widely accepted underlying mechanism. Ankylosis typically develops after the tooth has reached occlusal contact and may impair vertical alveolar bone growth, resulting in infraocclusion. Another commonly proposed factor is the congenital absence of permanent successors. Additional aetiological factors include local mechanical trauma, chemical or thermal irritation, abnormal eruption paths of permanent successors, excessive tongue pressure, and certain systemic conditions, such as congenital syphilis [7]. A genetic predisposition has also been suggested, based on the higher prevalence reported among siblings and monozygotic twins; however, no specific gene has yet been conclusively associated with infraocclusion [8,9].

Clinically, infraocclusion commonly presents with tipping of adjacent teeth and supraeruption of opposing teeth. Reduced cleanability may increase the risk of caries, and infraocclusion can also lead to delayed eruption or altered eruption paths of permanent successors, potentially contributing to malocclusion. Radiographically, infraocclusion may be associated with vertical discrepancies in the occlusal plane, interproximal alveolar bone sloping, and narrowing or loss of the periodontal ligament space in ankylosed cases. Percussion of ankylosed teeth may produce a metallic sound; however, definitive diagnosis may require advanced imaging, as ankylosis can be microscopic and not always radiographically evident [10,11,12].

Infraocclusion frequently coexists with dental anomalies, including hypodontia, supernumerary teeth, ectopic eruption of permanent molars, peg-shaped lateral incisors, palatally displaced maxillary canines, and enamel hypoplasia. Additional anomalies such as tooth agenesis, microdontia, delayed tooth development, fusion, taurodontism, dens invaginatus, talon cusp, and radix entomolaris have also been reported [13]. These anomalies appear more frequently in individuals with infraocclusion than in the general population, forming dental anomaly patterns (DAP), which suggest that infraocclusion may present a component of broader developmental disturbances rather than an isolated finding [14].

Treatment planning for infraoccluded primary molars should be individualized, considering factors like timing of diagnosis, progression rate, patient age, severity of infraocclusion, presence of ankylosis, type of malocclusion, and presence of permanent successors. The primary objectives of treatment include maintaining arch space, guiding eruption of permanent teeth, and reducing complications such as adjacent tooth tipping, antagonist supraeruption, and alveolar bone defects. Conservative management, including monitoring or restorative approaches, may be appropriate in mild cases, particularly when permanent successors are present. In contrast, severe cases without successors or with a lack of alveolar bone development may require extraction, space maintenance, or decoronation to preserve alveolar ridge integrity for future prosthetic rehabilitation. Early diagnosis and timely intervention are therefore important to minimize complications during the mixed dentition period [15].

Previous studies have examined infraocclusion in primary molars from different perspectives. For instance, Bjerklin and Bennett longitudinally evaluated root resorption and tipping of adjacent teeth using intraoral radiographs [16]. Peretz et al. investigated the prevalence of infraocclusion in mandibular primary molars, its association with alveolar bone height, and adjacent tooth movement [17]. Díaz Schiappacasse et al. assessed infraocclusion through clinical examinations, categorizing cases according to gender, location, number of affected teeth, severity, and unilateral or bilateral occurrence [18]. Răducanu and Feraru [19] evaluated the prevalence, severity, complications, and treatment approaches, whereas Salem and Mirzaee focused specifically on prevalence and associated dental anomalies [20]. Naiboğlu and Aktören analyzed prevalence, gender and age distribution, presence of permanent successors, and clinical features of affected teeth [21].

Although these studies have provided valuable insights into different aspects of infraocclusion, most have examined limited variables or involved relatively small sample sizes. Therefore, there remains a need for more comprehensive investigations that simultaneously evaluate multiple clinical and radiographic parameters. In this study, infraocclusion was investigated as a condition in which primary molars are positioned below the occlusal plane, independent of the underlying pathological etiology. Accordingly, this study aimed to determine the prevalence of infraocclusion in primary molars, its distribution according to age, gender, and location, associated dental anomalies, relevant radiographic findings, and the treatments applied to affected teeth.

## 2. Materials and Methods

This retrospective study was conducted at the Department of Paediatric Dentistry, Faculty of Dentistry, Gazi University. Digital panoramic radiographs (DPRs) of children aged 7–11 years, routinely obtained as part of standard clinical care between January 2019 and December 2022, were retrospectively reviewed. The study protocol was approved by the Ethics Committee of Gazi University (approval date: 27 February 2024; approval number: E-77082166-302.08.01-897993). Informed consent forms signed by parents or legal guardians were obtained prior to the use of patient data, and all patient data were anonymized before analysis

### 2.1. Sample Size

The minimum required sample size for this retrospective study was calculated using the formula recommended by Daniel (2018) for finite populations, with a 95% confidence level and a 5% margin of error [22]. Based on a total population of 8761 DPRs and an estimated infraocclusion prevalence of 3.5%, the minimum sample size was determined to be 51. Initially, a total of 8761 DPRs were screened. After applying inclusion and exclusion criteria, 8452 radiographs were ultimately included.

### 2.2. Inclusion and Exclusion Criteria

The inclusion criteria were as follows: healthy children aged 7–11 years, with health status determined based on routinely recorded clinical examination and anamnesis data available in the electronic patient records, whose first permanent molars had reached full occlusion, and DPRs with adequate image quality allowing diagnostic evaluation of the entire jaw. Children were excluded from the study if they presented systemic conditions affecting dental development, primary molars with excessive structural loss, partially erupted or unerupted first permanent molars, absence of adjacent teeth necessary for determining infraocclusion, or DPRs with poor image quality—such as blurriness, superimpositions, exposure errors, or artifacts—that compromised diagnostic interpretation.

### 2.3. Data Collection

All radiographs were retrieved from the Picture Archiving and Communication System (PACS) of the Gazi University Faculty of Dentistry Patient Information Management System. DPRs included in the study were obtained at the time of the initial clinical examination as part of routine dental assessment. Patient identifiers were removed, and only anonymized data were analyzed. DPRs were reviewed independently by two research assistants from the Department of Pediatric Dentistry, each possessing three years of clinical and radiographic diagnostic experience, using an LCD monitor (ASUS, Taipei, Taiwan). Interobserver reliability was high (κ = 0.98).

Additionally, treatments applied to primary molars with infraocclusion were systematically recorded. All treatment data were retrieved from the Metasoft DentAssist digital database system, documenting clinical procedures alongside the corresponding tooth number and application date. For each infraoccluded tooth identified on DPRs, only treatment records dated after radiographic examination were considered. Accordingly, treatment categories—derived from the institutional digital patient record system—reflect documented clinical management at the time of care rather than inferred indications, due to the retrospective design of the study.

### 2.4. Evaluation Criteria for Infraocclusion

Infraocclusion was identified and recorded for each primary molar according to the following parameters:

- Distribution by gender, dental arch (maxilla/mandible), and tooth type (first/second molar).
- Presence or absence of permanent successors.
- Associated dental anomalies (e.g., hypodontia, supernumerary teeth, taurodontism identified in any region of the maxilla or mandible).
- Radiographic findings including adjacent tooth tipping, supraeruption of opposing teeth, presence of dental caries, and eruption disturbances in permanent successors.
- Treatment approach categorized as no treatment, preventive (i.e., fissure sealant application), restorative (composite resin, compomer, amalgam, and glass ionomer cement restorations), stainless steel crowns, or extraction.

Severity of infraocclusion was categorized using the Brearley and McKibben classification system [6]:

- Mild: Occlusal surface approximately 1 mm below the occlusal plane.
- Moderate: Occlusal surface approximately at the level of the interproximal contact points of one or both adjacent tooth surfaces.
- Severe: Occlusal surface at or below the interproximal gingival level of neighboring tooth surfaces.

### 2.5. Statistical Analysis

Statistical analyses were conducted using SPSS version 28.0 (Statistical Package for Social Sciences). Descriptive statistical methods (number, percentage) were employed to summarize data. The chi-square test was used to evaluate statistically significant relationships between categorical variables. A *p*-value of less than 0.05 (α = 0.05) was considered statistically significant for all analyses.

## 3. Results

Infraocclusion was detected in one or more primary molars in 305 patients. There was no statistically significant association between gender and infraocclusion (*p* > 0.05) (Table 1). Among these 305 cases, unilateral involvement was observed in 207 patients (67.9%), and bilateral involvement was observed in 98 patients (32.1%).

A total of 22.2% of primary molars with infraocclusion were in the maxilla, whereas 77.8% were in the mandible, meaning that infraocclusion was 4.47 times more frequent in the mandible than in the maxilla. The mandibular second molar was the most commonly affected tooth, though there was no statistically significant relationship between location, tooth type, and infraocclusion occurrence (*p* > 0.05).

Many patients had more than one affected tooth. Among the 305 patients with infraocclusion, 179 (58.7%) had 1 tooth affected, 85 (27.9%) had 2 teeth, 20 (6.6%) had 3 teeth, and 21 (6.9%) had 4 or more teeth affected. The mean number of infraoccluded teeth per patient was 1.7.

**Figure A1:**
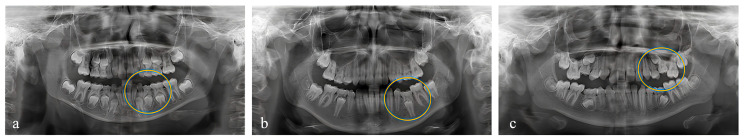
Panoramic radiographs of patients showing different levels of infraocclusion severity: (**a**) mild; (**b**) moderate; (**c**) severe.

The distribution of infraocclusion severity according to tooth type is presented in Table 2. Although no statistically significant association was found in the maxillary primary molars (*p* > 0.05), a significant relationship was observed in the mandibular primary molars (*p* < 0.05). Severe infraocclusion was significantly less common than moderate or mild infraocclusion in the mandible. Figure A1 (Appendix B) illustrates representative panoramic radiographs showing mild, moderate, and severe infraocclusion.

Infraocclusion was most common in the 9-year-old age group. Until age 9, the most commonly affected teeth were the first primary molars; however, at age 9, the second primary molars became more frequently affected. According to the statistical analysis, a significant relationship was found between age and affected tooth type (*p* < 0.05). Infraocclusion prevalence was significantly higher in the 9-year-old age group compared to other age groups (Table 3).

In 53 (10.3%) of the 513 infraoccluded teeth, the underlying permanent successor was absent. Congenital premolar agenesis was most common (43.4%) in the mild infraocclusion group. No statistically significant relationship was found between the presence of permanent successors and infraocclusion severity (*p* > 0.05) (Table 4).

Among the 305 patients with infraocclusion, 154 (50.5%) exhibited associated dental anomalies. Dens invaginatus was identified in 33.1% and hypodontia in 32.5% of the patients. These were followed by supernumerary teeth (9.1%), taurodontism (7.8%), mesiodens (4.5%), oligodontia (3.2%), radix entomolaris (3.2%), odontoma (2.6%), peg-shaped lateral (2.0%) and talon cusps (2.0%).

The relationship between infraocclusion severity and accompanying radiographic findings was analyzed using the Chi-square test. As presented in Table 5, statistically significant associations were identified between infraocclusion severity and adjacent tooth tipping, supraeruption of the opposing teeth, and eruption problems of permanent successors (*p* < 0.05). However, no significant relationship was found regarding dental caries (*p* > 0.05).

There was also a statistically significant association between infraocclusion severity and treatment modality applied, as evaluated by the Chi-square test (*p* < 0.05; Table 6). In the severe infraocclusion group, restorative treatments were significantly less frequent, whereas extractions were significantly more frequent compared to other groups.

Among infraoccluded teeth, 252 (54.8%) of the 460 teeth with permanent successors received no treatment, whereas 21 (39.6%) of the 53 teeth without permanent successors were untreated. Analysis indicated a statistically significant relationship between the presence of permanent successors and treatment modality (*p* < 0.05) (Table 7). Stainless steel crowns were significantly more common in teeth without permanent successors (11.3%) compared to those with successors (0.7%). Similarly, extractions were significantly more common when successors were present, whereas preventive treatments were more frequent in the absence of permanent successors.

## 4. Discussion

In the literature, terms such as ankylosis, submerged teeth, secondary retention, and reinclusion have often been used interchangeably to describe teeth positioned below the occlusal plane, despite representing distinct clinical entities with different etiologies and pathophysiological mechanisms [2,23,24]. Although these conditions may share a similar clinical appearance, they should not be considered equivalent. Such terminological diversity may complicate comparisons across studies. In the present study, ‘‘infraocclusion’’ was used consistently, as it descriptively refers to the relative vertical position of the affected tooth in relation to adjacent teeth, irrespective of etiology or severity.

The study sample primarily consisted of children who attended the Paediatric Dentistry Clinic for routine dental examination and general dental care. Literature detailing radiographic findings and treatment modalities associated with primary molar infraocclusion in Turkish children remains limited [21,25,26]. Most previous studies have included relatively small sample sizes and focused on a restricted number of variables. A key strength of the present study is its larger sample size and comprehensive evaluation of infraocclusion prevalence, radiographic characteristics, associated dental anomalies, and treatment modalities.

Previous studies have reported a wide range of prevalence values for infraocclusion in primary molars [18,19,20,21,25,26,27,28,29]. The prevalence observed in the present study (3.6%) is consistent with findings reported in other Turkish cohorts [21,25,26]. This variability may be related to differences in study design, diagnostic criteria, age distribution, and inclusion or exclusion criteria rather than sample size alone. The inclusion of different age groups may directly influence reported prevalence rates. In older children, clinically more severe cases are more likely to be detected, whereas mild infraocclusion may be overlooked, particularly due to exfoliation or reduced clinical visibility. In addition, mild infraocclusion may be identified more accurately through detailed clinical examination and periapical radiographs compared with panoramic radiographs alone. Furthermore, inconsistent definitions of infraocclusion in the literature—often overlapping with terms such as ankylosis or secondary retention—may have led researchers to classify conditions other than true infraocclusion as infraocclusion, thereby contributing to variability in prevalence findings.

In the present study, no statistically significant association was found between gender and infraocclusion, which is consistent with several previous studies reporting similar frequencies in male and female patients [9,21,25,26,30]. However, some studies have reported a higher prevalence in males [31].

Several studies have reported that infraocclusion predominantly presents bilaterally [21,32]. However, Raducanu and Feraru reported asymmetric involvement in the maxilla and predominantly symmetric involvement in the mandible [19]. In contrast, unilateral involvement was more common in the present study, which is consistent with the findings of Alshaya et al. [31]. Although unilateral infraocclusion appears to be more frequent, the possibility of multiple teeth being affected in the same patient should be considered. Therefore, when an infraoccluded primary molar is identified, careful evaluation of other primary molars may be warranted.

Certain studies have reported mandibular first molars as the most commonly affected teeth [19,20,26,33], whereas others have identified mandibular second molars as the most frequently involved [18,25,31,34]. In the present study, mandibular second primary molars were the teeth most commonly affected by infraocclusion. Discrepancies among studies may be explained by the tendency of primary molars to exhibit mild infraocclusion, which may remain undiagnosed due to spontaneous exfoliation [35].

Prevalence studies examining which tooth type is more frequently affected by infraocclusion across different age groups have indicated variability, yet data remain insufficient. In the present study, as shown in Table 3, mandibular primary molars—particularly the first primary molars in children under 9 years of age—were more frequently affected by infraocclusion. Consistent with our findings, Kurol reported that mandibular first primary molars were the most commonly affected teeth in children younger than 9 years, whereas infraocclusion of second primary molars predominated after this age [4]. This pattern may be explained by the earlier development of first molars and their greater susceptibility to environmental factors. In contrast, after the age of 9 years, increased masticatory forces have been suggested to raise the risk of infraocclusion in second molars [36].

Shalish et al. identified a significant relationship between infraocclusion and anomalies such as dental agenesis, microdontia of maxillary lateral incisors, palatally displaced canines, and distal tipping of mandibular second premolars [37]. Similarly, Venza et al. reported associations with impacted teeth, palatally displaced maxillary canines, and supernumerary teeth [29]. Conversely, the same study did not find statistically significant associations between infraocclusion and hypodontia, dental transposition, or odontomas, contrary to previous literature. In contrast, Lochib et al. found no association between infraocclusion and dental anomalies in preschool children aged 3–5 years [38]. The associations between infraocclusion and other dental anomalies reported in the literature, along with our findings, suggest a possible shared genetic origin. Given the retrospective and cross-sectional nature of the available data, infraocclusion cannot be regarded as a confirmed clinical early indicator of other dental anomalies; however, it may be hypothesised that infraocclusion could represent an early sign of certain associated anomalies, a possibility that warrants further longitudinal investigation.

In studies evaluating infraocclusion based on clinical or radiographic findings, tipping of adjacent teeth has been reported as the most frequent associated finding [34]. Shalish et al. reported that among25 orthodontic patients with at least one severely infraoccluded primary molar, severe tipping of adjacent teeth and impacted premolars were observed in all cases [37]. Similarly, previous studies have reported additional radiographic findings such as midline deviation, increased caries, root resorption, and supraeruption of the opposing tooth [26]. Consistent with these reports, our findings indicate that the frequency and severity of associated radiographic changes increase with the severity of infraocclusion. In particular, statistically significant associations were observed between infraocclusion severity and adjacent tooth tipping, supraeruption of the opposing tooth, and eruption problems of permanent successors (*p* < 0.05), whereas no significant association was found with dental caries.

Although many case reports describe treatments applied to infraoccluded teeth, retrospective studies evaluating clinician approaches to managing these teeth in broader patient populations presenting at clinics remain limited. In the present study, conservative approaches—including observation, preventive measures, and restorative treatments—were more commonly applied in cases of mild infraocclusion, whereas extraction was more frequently preferred in severe cases. Furthermore, stainless steel crowns were more commonly placed in patients without permanent successors compared to those with permanent successors, indicating a preference for crowns when permanent successors were absent. Similar treatment patterns were reported by Akgöl et al., where restorative treatment was favoured in mild cases and extraction in severe cases [26]. These findings indicate that treatment strategies become increasingly invasive as infraocclusion severity progresses. From a clinical perspective, fissure sealant application can be considered a conservative preventive approach for infraoccluded primary molars during the mixed dentition period. Due to altered occlusal contacts and reduced self-cleansing, these teeth may be more susceptible to caries. Therefore, fissure sealants may help preserve tooth structure and reduce the need for more invasive treatments in mild cases.

Several studies have reported that primary molars with infraocclusion are frequently ankylosed [34,39]. Although two-dimensional imaging methods such as intraoral radiographs or DPRs, are considered insufficient for the definitive diagnosis of ankylosis, cone-beam computed tomography (CBCT) is recognized as reliable. Nevertheless, supplementary clinical findings remain essential for a conclusive diagnosis [40]. In the present study, infraoccluded primary molars were identified via DPRs, and no clinical evaluation was conducted. Consequently, it is not possible to definitively ascertain whether infraoccluded primary molars identified only via DPRs and without clinical examination were ankylosed.

This study has several limitations. First, high-quality digital panoramic radiographs (DPRs) with minimal superimposition and distortion were used to accurately identify infraocclusion and assess its severity. The visual assessment of severity without quantitative measurements may be considered a limitation of the study. Although periapical radiographs could have been used to assess infraocclusion severity, periapical images in retrospective datasets are often region-specific and may not be available for bilateral comparison. In contrast, DPRs were preferred over periapical radiographs because they provide standardized bilateral visualization of the dentition, which is essential for evaluating unilateral versus bilateral involvement. Another limitation is related to the retrospective nature of the study, as it was not possible to determine whether infraoccluded teeth were treated or extracted primarily because of caries or specifically to minimise the clinical consequences of infraocclusion. This uncertainty may affect the accurate interpretation of the study findings.

When all the data from our study were analyzed, the observed prevalence of infraocclusion was comparable to that reported by researchers employing large sample sizes, supporting findings from other populations regarding age distribution, affected tooth type, location, and presence of permanent successor regression. This study may serve as a valuable reference in enhancing awareness of the presence or absence of permanent successors beneath infraoccluded primary molars, assisting clinicians in selecting appropriate treatment options. By elucidating the clinical and radiographic features associated with infraocclusion, the findings of this study could significantly contribute to pediatric dentists’ diagnostic and treatment planning processes.

Future studies should focus on further clarifying infraocclusion using advanced imaging techniques such as CBCT, which can facilitate more precise assessments, particularly in suspected ankylosis cases. Additionally, investigating genetic and environmental factors contributing to infraocclusion, conducting long-term follow-up studies to assess its progression and effects on malocclusion and jaw development, and comparing the success rates of various treatment modalities could substantially enrich current knowledge. These research efforts would provide a more comprehensive understanding of the etiology, clinical implications, and management strategies for infraocclusion and contribute to the development of evidence-based treatment protocols.

## 5. Conclusions

Although infraocclusion of primary molars is relatively uncommon, it represents a clinically meaningful condition with potential implications for permanent successor development and overall occlusal integrity. The presence or absence of the permanent successor should be carefully evaluated, as it significantly influences both treatment planning and long-term outcomes. Given the progressive nature of its clinical effects, management of infraocclusion should be individualized and primarily guided by severity, with conservative approaches reserved for mild cases and more invasive interventions considered for severe presentations to prevent occlusal and eruption-related complications.

## Figures and Tables

**Table 1:** Distribution of infraocclusion by gender.

		Infraocclusion		Total n (%)	X^2^	*p*
		Present	Absent			
		n (%)	n (%)			
Gender	Female	149 (3.4)	4233 (96.6)	4382 (100.0)	1.136	0.287
	Male	156 (3.8)	3914 (96.2)	4070 (100.0)		
	Total	305 (3.6)	8147 (96.4)	8452 (100.0)		

X^2^: Chi-square test.

**Table 2:** Distribution of infraocclusion severity by tooth.

Localization	Tooth	Severity of Infraocclusion				X^2^	*p*
		Mild	Moderate	Severe	Total		
		n (%)	n (%)	n (%)	n (%)		
Maxilla	1st primary molar	21 (51.2)	3 (7.3)	17 (41.5)	41 (100.0%)	5.794	0.055
	2nd primary molar	26 (35.6)	17 (23.3)	30 (41.1)	73 (100.0%)		
Mandible	1st primary molar	100 (57.1)	61 (34.9)	14 (8.0)	175 (100.0%)	11.407	0.003 *
	2nd primary molar	107 (47.8)	72 (32.1)	45 (20.1)	224 (100.0%)		
	Total	254 (49.5)	153 (29.8)	106 (20.7)	513 (100.0%)		

* *p* < 0.05, X^2^: Chi-square test.

**Table 3:** Distribution of primary molars with infraocclusion according to age.

		Tooth		Total (%)	X^2^	*p*
		1st Primary Molar	2nd Primary Molar			
		n (%)	n (%)			
Age	7	35 ^a^ (70.0)	15 ^b^ (30.0)	50 (100.0)	57.903	0.000 *
	8	64 ^a^ (57.1)	48 ^b^ (42.9)	112 (100.0)		
	9	65 ^a^ (46.1)	76 ^b^ (53.9)	141 (100.0)		
	10	35 ^a^ (31.8)	75 ^b^ (68.2)	110 (100.0)		
	11	17 ^a^ (17.0)	83 ^b^ (83.0)	100 (100.0)		
	Total	216 (42.5)	297 (57.5)	513 (100.0)		

* *p* < 0.05, X^2^: Chi-square test. Different superscript letters (^a^, ^b^) within the same row indicate which groups differ significantly from each other.

**Table 4:** Distribution of infraocclusion severity according to the presence of a permanent successor.

	Severity of Infraocclusion			Total n (%)	X^2^	*p*
	Mild	Moderate	Severe			
	n (%)	n (%)	n (%)			
Infraoccluded primary molars with successors	231 (50.2)	134 (29.1)	95 (20.7)	460 (100.0)	1.166	0.558
Infraoccluded primary molars without successors	23 (43.4)	19 (35.8)	11 (20.8)	53 (100.0)		
Total	254 (49.5)	153 (29.8)	106 (20.7)	513 (100.0)		

X^2^: Chi-square test.

**Table 5:** Relationship between infraocclusion severity and radiographic findings.

Accompanying Radiographic Findings	Severity of Infraocclusion			Total n (%)	X^2^	*p*
	Mild	Moderate	Severe			
	n (%)	n (%)	n (%)			
Tipping of adjacent teeth	53 (21.3)	97 (39.0)	99 (39.8)	249 (100.0)	176.785	0.000 *
Supraeruption of opposing tooth	35 (30.4)	46 (40.0)	34 (29.6)	115 (100.0)	21.728	0.000 *
Presence of dental caries	158 (50.2)	92 (29.2)	65 (20.6)	315 (100.0)	0.174	0.917
Problems with permanent tooth eruption	6 (12.2)	9 (18.4)	34 (69.4)	49 (100.0)	79.827	0.000 *

* *p* < 0.05, X^2^: Chi-square test.

**Table 6:** Relationship between infraocclusion severity and treatment modalities applied.

	Severity of Infraocclusion			Total n (%)	X^2^	*p*
	Mild	Moderate	Severe			
Treatment Applied	n (%)	n (%)	n (%)			
No treatment	139 ^a^ (54.7)	82 ^a^ (53.6)	52 ^a^ (49.1)	273 (53.2)	50.320	0.000 *
Restorative treatment	61 ^a^ (24.0)	26 ^a,b^ (17.0)	8 ^b^ (7.5)	95 (18.5)		
Stainless steel crowns	4 ^a^ (1.6)	3 ^a^ (2.0)	2 ^a^ (1.9)	9 (1.8)		
Extraction	31 ^a^ (12.2)	27 ^a^ (17.6)	43 ^b^ (40.6)	101 (19.7)		
Preventive treatment	19 ^a^ (7.5)	15 ^a^ (9.8)	1 ^b^ (0.9)	35 (6.8)		
Total	254 (100.0)	153 (00.0)	106 (100.0)	513 (100.0)		

* *p* < 0.05, X^2^: Chi-square test. Different superscript letters (^a^, ^b^) within the same row indicate which groups differ significantly from each other.

**Table 7:** Relationship between treatment modality and presence of permanent successors in infraoccluded primary molars.

	Permanent Successor		Total n (%)	X^2^	*p*
	Present	Absent			
Treatment Applied	n (%)	n (%)			
No treatment	252 ^a^ (54.8)	21 ^b^ (39.6)	273 (53.2)	48.770	0.000 *
Restorative treatment	84 ^a^ (18.3)	11 ^a^ (20.8)	95 (18.5)		
Stainless steel crowns	3 ^a^ (0.7)	6 ^b^ (11.3)	9 (1.8)		
Extraction	96 ^a^ (20.9)	5 ^b^ (9.4)	101 (19.7)		
Preventive treatment	25 ^a^ (5.4)	10 ^b^ (18.9)	35 (6.8)		
Total	460 (100.0)	53 (100.0)	513 (100.0)		

* *p* < 0.05, X^2^: Chi-square test. Different superscript letters (^a^, ^b^) within the same row indicate which groups differ significantly from each other.

## Data Availability

The original contributions presented in this study are included in the article. Further inquiries can be directed to the corresponding author.

## Abbreviations

DAP	Dental anomaly patterns
DPRs	Digital panoramic radiographs
CBCT	Cone beam computed tomography

## Appendix A

**Table A1:** Reported prevalence of infraocclusion and the most commonly affected teeth in the literature.

Author	Country	Year	Prevalence (%)	Most Commonly Affected Tooth
Zúñiga-Tertre et al. [27]	Spain	2004	10.48	Mandibular 1st primary molar
Rãducanu and Feraru [19]	Romania	2005	7.16	Mandibular 1st primary molar
Salem and Mirzaee [20]	Iran	2009	15.0	Mandibular 1st primary molar
Silvestrini Biavati et al. [28]	Italy	2011	6.6	Mandibular 2nd primary molar
Cardoso et al. [33]	Spain	2014	21.8	Mandibular 1st primary molar
Venza et al. [29]	Italy	2018	2.8	Mandibular 2nd primary molar
Díaz Schiappacasse et al. [18]	Chile	2019	41.78	Mandibular 2nd primary molar
Çiftçi et al. [25]	Turkey	2021	3.84	Mandibular 2nd primary molar
Naiboğlu and Aktören [21]	Turkey	2022	3.6	Mandibular 2nd primary molar
Alshaya et al. [31]	Saudi Arabia	2022	7.38	Mandibular 2nd primary molar
Akgöl et al. [26]	Turkey	2024	4.3	Mandibular 1st primary molar
Kim et al. [5]	Republic of Korea	2024	13.0	Mandibular 1st primary molar
